# Lymphocyte-activation gene 3 in cancer immunotherapy: function, prognostic biomarker and therapeutic potentials

**DOI:** 10.3389/fimmu.2024.1501613

**Published:** 2024-11-26

**Authors:** Ke Ren, Hayam Hamdy, Abdo Meyiah, Eyad Elkord

**Affiliations:** ^1^ Department of Biosciences and Bioinformatics, School of Science, Suzhou Municipal Key Lab in Biomedical Sciences and Translational Immunology, Xi’an Jiaotong-Liverpool University, Suzhou, Jiangsu, China; ^2^ Department of Forensic Medicine and Toxicology, Faculty of Veterinary Medicine, New Valley University, New Valley, Egypt; ^3^ College of Health Sciences, Abu Dhabi University, Abu Dhabi, United Arab Emirates; ^4^ Biomedical Research Center, School of Science, Engineering and Environment, University of Salford, Manchester, United Kingdom

**Keywords:** biomarker, T cells, LAG-3, immune checkpoint, cancer

## Abstract

Lymphocyte-activation gene 3 (LAG-3) has emerged as a key immune checkpoint regulating immune responses in the context of cancer. The inhibitory effect of LAG-3-expressing T cells contributes to suppressing anti-tumor immunity and promoting tumor progression. This review discusses the function of LAG-3 in immune suppression, its interactions with ligands, and its potential as a prognostic biomarker for cancers. We also explore therapeutic strategies targeting LAG-3, including monoclonal antibodies, small molecule inhibitors, and CAR T cells. This review summarizes the current preclinical and clinical studies on LAG-3, highlighting the potential of therapeutic regimens targeting LAG-3 to enhance antitumor immunity and improve patients’ outcomes. Further studies are needed to fully elucidate the mechanism of action of LAG-3 and optimize its application in tumor therapy.

## 1 Introduction

Despite significant advances in cancer treatment, cancer remains a leading cause of human death (1). Traditional treatments, such as chemotherapy and radiotherapy, often have limited therapeutic effects due to adverse reactions and acquired drug resistance (2). Therefore, new treatment options are constantly explored, among which immune checkpoint inhibitors (ICIs) have brought breakthroughs in tumor treatment strategies.

ICIs enhance anti-tumor immune responses by blocking immune checkpoint proteins that inhibit T cell function, such as cytotoxic T lymphocyte antigen 4 (CTLA-4) and programmed death protein 1 (PD-1) (1). In March 2011, the US Food and Drug Administration (FDA) approved ipilimumab (Yervoy^®^), the first anti-CTLA-4 monoclonal antibody (mAb), for the treatment of advanced melanoma (3–5). Subsequently, PD-1 and PD-L1 inhibitors were approved and showed significant clinical effects in various cancers, prolonging patients’ progression-free survival (PFS) and overall survival (OS) (6, 7). However, many cancer patients have limited responses to mAbs, and this therapy still causes immune-related adverse effects (irAEs) such as autoimmune dermatitis, colitis, and endocrine diseases (8, 9).

Researchers are searching for novel immune checkpoint targets to improve the efficacy and safety of ICI therapy. In this context, Lymphocyte-activation gene 3 (LAG-3), an emerging immune checkpoint protein, has attracted additional attention. LAG-3 has a similar structure to CD4 and is expressed on the surface of various immune cells, including activated T cells, T regulatory cells (Tregs) and natural killer cells (NK cells) (10–15). As an inhibitory immune checkpoint, LAG-3 suppresses excessive immune responses in the immune system by inhibiting the activation and proliferation of T cells, thereby reducing the risk of immune system attack on autologous tissue (16, 17). This function particularly depends on the interaction of LAG-3 expressed on Tregs and ligands on antigen-presenting cells (APCs) (18). However, in the tumor microenvironment (TME), tumor cells can escape immune surveillance by hijacking this mechanism (19–21).

Recent studies have shown that LAG-3 can be co-expressed with other immune checkpoints, leading to T cell exhaustion (22–24). Exhausted T cells have a weakened ability to produce cytokines and reduced cytotoxicity, resulting in inhibition of their ability to target and clear tumor cells (25, 26). Anti-LAG-3 mAbs have shown the capacity to restore T cell function and alleviate T cell exhaustion (27). In addition, the high expression of LAG-3 in a variety of tumors is correlated with prognosis of cancer patients, which shows its potential as an independent prognostic biomarker for tumor treatment (28).

This review discusses the function of LAG-3 in regulating immune responses, the effect of LAG-3 interaction with ligands, the significance of LAG-3 as a prognostic biomarker in cancer patients and the potential therapeutic strategies targeting LAG-3.

## 2 Functions of LAG-3 in regulating immune responses

LAG-3 is a critical protein controlling immune responses and promoting growth of tumors (29). Through antibody-blocking experiments on the mouse and human cells, it was found that LAG-3 maintained immune homeostasis by negatively regulating the proliferation, activation, and effector function of CD4^+^ and CD8^+^ T cells, thereby avoiding tissue damage and autoimmune complications (12, 13, 30, 31). However, in the TME, tumor cells often use this inhibitory function of LAG-3 to evade the immune system’s surveillance (20). Tumor-infiltrating lymphocytes (TILs) expressing high levels of LAG-3 are usually dysfunctional, characterized by decreased proliferation and decreased ability to secrete cytokines such as IL-2, TNF-α and IFN-γ (24, 32, 33).

LAG-3 also affects Tregs, which are critical for maintaining immune tolerance and preventing autoimmune diseases (29, 34). Tregs inhibit the activation and proliferation of potentially autoreactive T cells in the healthy body by active regulation, thereby maintaining immune homeostasis (35). In the TME, the activation of LAG-3 can enhance the function of Tregs and inhibit the clearance of tumor cells by the immune system, thereby promoting tumor immune escape (34, 36). In addition, LAG-3 regulates the function of other immune cells, including natural killer cells (NK cells) and plasmacytoid dendritic cells (pDCs) (37). LAG-3 regulates innate immune responses by attenuating NK cells’ cytotoxic activity and cytokine production by interacting with its ligands (38). In pDCs, an increase in LAG-3 expression can inhibit the production of type I interferon (IFN-1), which is a critical factor in initiating antiviral immune responses (39).

The inhibitory signaling pathway of LAG-3 was also investigated. Holfman et al. indicated that LAG-3 and PD-1 inhibit T cell function through different pathways (40). They studied LAG-3 and PD-1 on exhausted CD8^+^ T cells and found that PD-1 limited T cell proliferation by inhibiting TCR and co-receptor signaling. LAG-3 pathway differs in that it inhibits cytokine production and cytotoxic activity, thereby inhibiting the ability of exhausted CD8^+^ T cells to kill target cells (40). This distinction allows for a synergistic effect of PD-1 and LAG-3 during T cell exhaustion. In addition, LAG-3 further promotes and maintains the exhausted state of T cells by maintaining TOX expression and regulating the NK receptor pathway on the T cell surface (41). The multiple functions of LAG-3 in the regulation of the immune system further emphasize its potential as a therapeutic target for autoimmune diseases and cancer.

## 3 Effect of the interaction of LAG-3 with ligands

### 3.1 MHC-II

**Figure 1:**
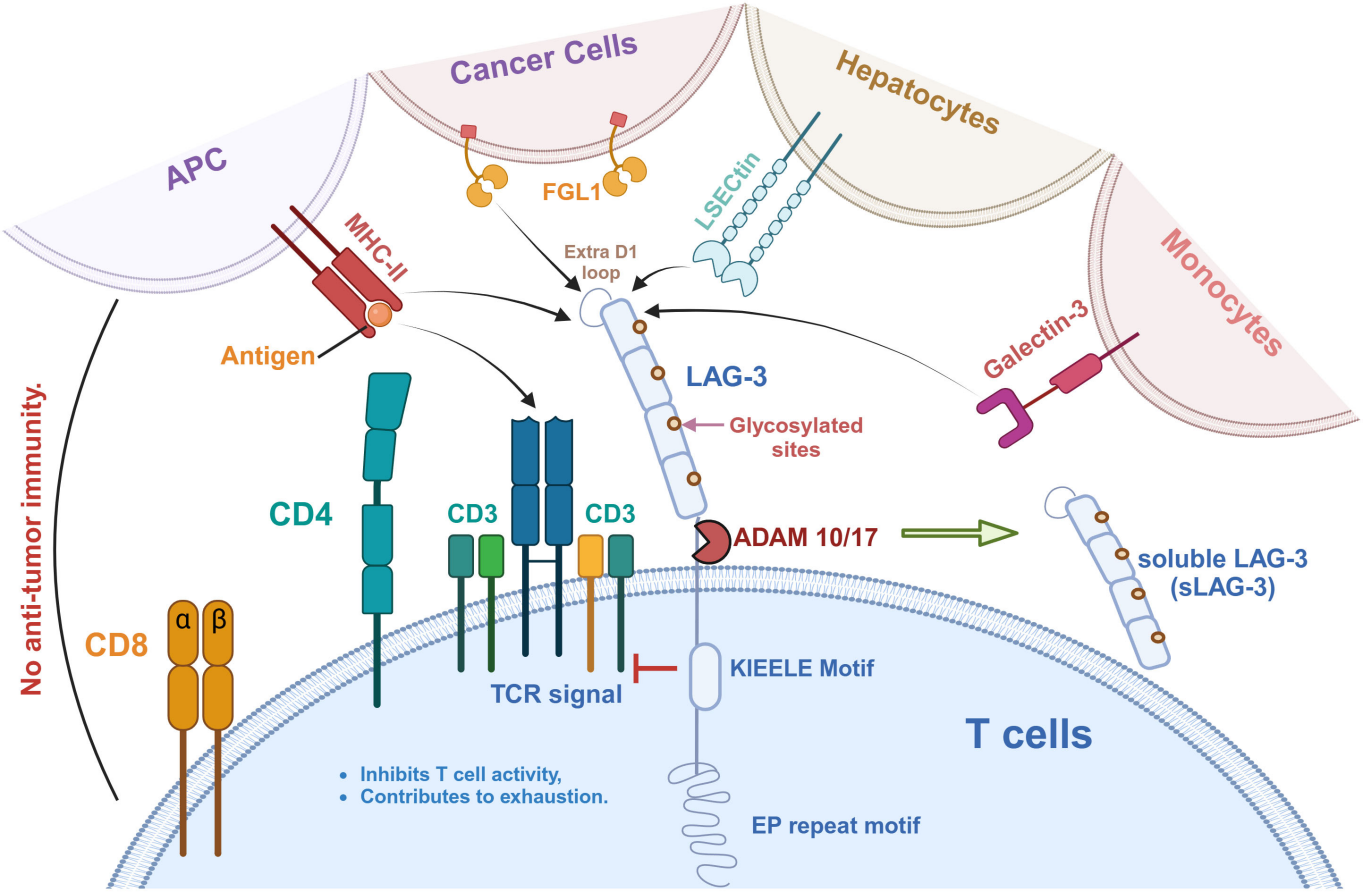
LAG-3 signaling pathway and its ligands in tumor immunity. LAG-3 is expressed on T cells and interacts with a variety of ligands to inhibit T cell activity and promote immune exhaustion. These ligands include MHC-II on antigen-presenting cells (APCs), FGL1 on cancer cells, LSECtin on hepatocytes, Galectin-3 on monocytes, and the recently identified TCR-CD3 complex. As shown, MHC-II binds to the extra loop of the D1 domain of LAG-3, whereas FGL1 binds to other part of D1. A large number of glycosylation sites is present on LAG-3, which is also the binding site of LSECtin and Galectin-3. Notably, the effect of LAG-3 on the TCR-CD3 complex is indirect, in which the KIEELE motif plays a key role in regulating TCR-CD3 function. In addition, LAG-3 can be hydrolyzed by ADAM10/17 to produce soluble LAG-3 (sLAG-3), which further regulates immune responses.

**Figure 2:**
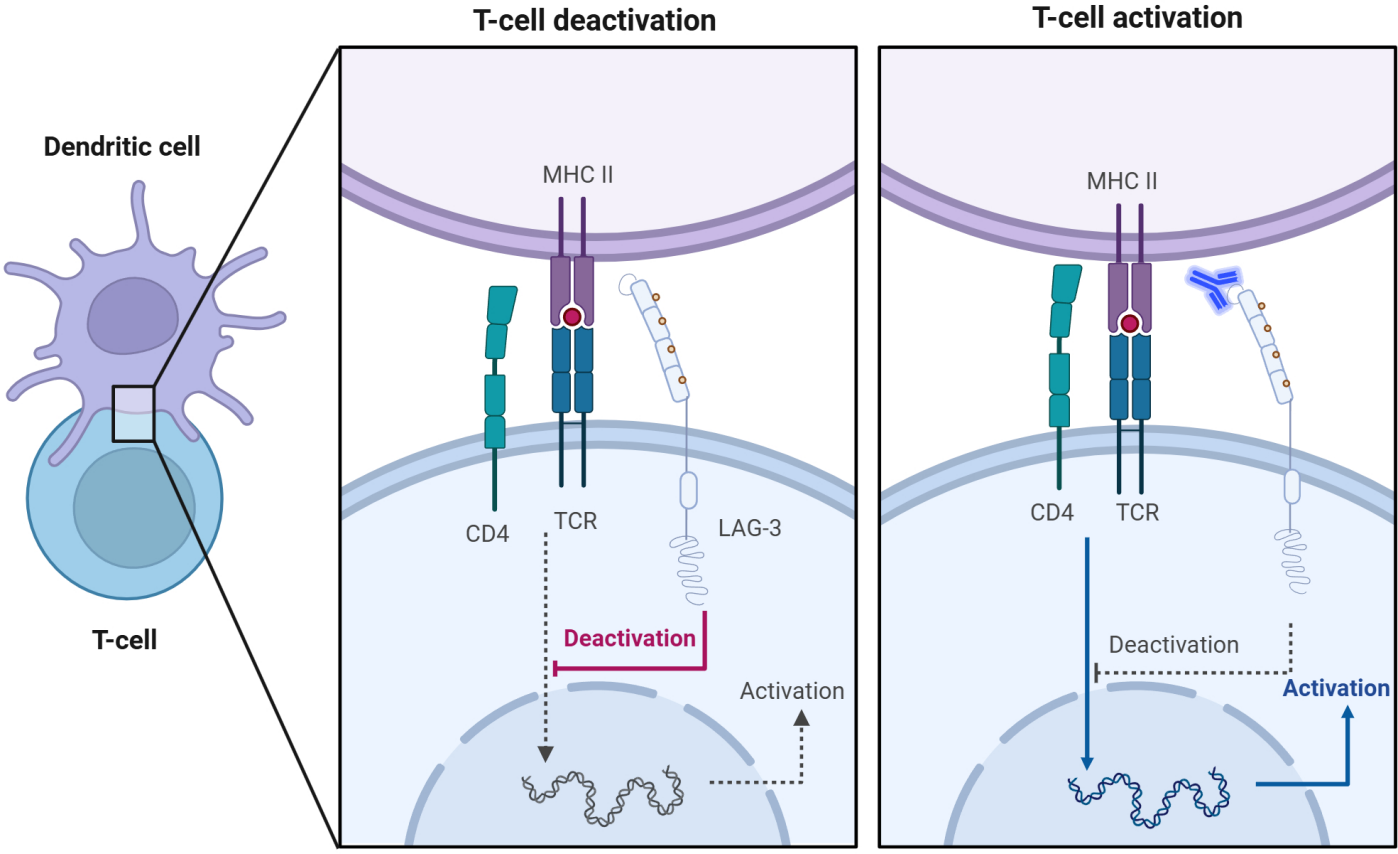
Anti-LAG-3 mAb restores T cell activation. Competitive binding of LAG-3 to MHC-II inhibits TCR signaling, leading to T cell inactivation (left). The addition of anti-LAG-3 mAbs blocked the binding of LAG-3 to MHC-II and restored the normal interaction between TCR and MHC-II, thereby promoting activation and proliferation of T cells (right).

LAG-3 is expressed on T cells and interacts with a variety of ligands to inhibit T cell activity, as shown in **Figure 1**. MHC-II is a natural ligand for LAG-3, and their binding has an essential effect on the behavior of T cells and tumor cells (42). On the one hand, the binding of LAG-3 to MHC-II affects anti-tumor immune responses by negatively regulating the activity of T cells (42, 43). In detail, LAG-3 binds to MHC-II molecules with high affinity and blocks T cell receptor (TCR) signaling, thereby inhibiting T cell proliferation, activation, and cytokine secretion (32, 33) (**Figure 2**). This inhibitory effect not only affects CD4^+^ and CD8^+^ T cells but also promotes the expansion of Tregs in the TME, which have a stronger immunosuppressive function and can further limit anti-tumor immune responses through cell contact (35, 44).

On the other hand, interaction of LAG-3 with MHC-II activates several survival pathways within tumor cells, including MAPK/Erk and PI3K/Akt signaling pathways (45, 46). Activating these pathways enhances tumor cell resistance to apoptosis, especially in MHC-II-expressing melanoma cells (46). It has been shown that LAG-3 binding to MHC-II protects tumor cells from Fas-mediated apoptosis and drug-induced apoptosis, such as etoposide, by activating the MAPK/Erk pathway and the PI3K/Akt pathway (47).

Overall, the binding of LAG-3 to MHC-II affects immune escape mechanisms in two ways. On one hand, it inhibits the immune surveillance function of T cells, and on the other hand, it enhances the survival of tumor cells. This interaction makes tumor cells more resistant in the face of the host immune system and therapeutic interventions, suggesting that strategies targeting LAG-3 and MHC-II interaction may have substantial clinical applications in cancer therapy.

### 3.2 Galectin-3 (Gal-3)

Galectin-3 (Gal-3), or lectin-3, is a glycoprotein widely expressed in immune cells, including macrophages, dendritic cells, and NK cells (30) (**Figure 1**). Studies have shown that Gal-3 is able to induce T cell anergy through TCR aggregation, thereby inhibiting T cell function (48). This inhibition has been observed in a variety of cancers, such as endometrial cancer, vulvar squamous cell carcinoma, and multiple myeloma (49–51). Furthermore, the function of these inhibited T cells could be restored by the knockout of surface Gal-3. Kouo et al. found that LAG-3 expression in the TME is required for Gal-3 to exert its inhibitory function (52). In contrast to LAG-3^+^ CD8^+^ T cells, LAG-3 KO CD8^+^ T cells were able to secrete more IFN-γ in the presence of Gal-3 (52). These results suggest that the interaction of LAG-3 with Gal-3 is an essential mechanism for regulating CD8^+^ T cell function.

### 3.3 LSECtin

LSECtin is a C-type lectin-like receptor typically expressed in the liver and has inhibitory immunomodulatory effects (53) (**Figure 1**). Xu et al. found that LSECtin can be expressed on the surface of murine B16 melanoma cells, inhibiting tumor-specific T cell immune responses and promoting the growth of tumor cells (53). Blockade of LAG-3 significantly improved LSECtin-mediated IFN-γ secretion by CD8^+^ T cells (53). However, the study by Xu et al. only preliminarily demonstrated the effect of LSECtin and LAG-3 interaction on tumor cells. The specific mechanism and other functions still need to be further explored.

### 3.4 FGL-1

FGL-1 is another ligand for LAG-3, expressed in hepatocytes, tumor cells, and some immune cells (54) (**Figure 1**). FGL-1 secreted by tumor cells within the TME can bind to LAG-3 on TILs, thereby inhibiting the anti-tumor activity (55). An *in vivo* experiment by Wang et al. showed that anti-FGL-1 mAb effectively activated T cell function and enhanced anti-tumor effects in WT mice. However, in LAG-3-deficient mice, the addition of FGL-1 mAb had no effect on the activation of T cells (56). They suggested that the interaction between LAG-3 and FGL-1 was a key mechanism leading to T cell inhibition. When anti-FGL-1 antibody was added, the inhibitory signaling pathway was blocked and T cell function was restored in WT mice. However, the LAG-3 KO mice model lost its ability to suppress T cells, so that T cell activation did not change significantly after adding anti-FGL-1 antibody. Furthermore, FGL-1 showed a more significant inhibitory effect on 3A9 T cell lines with IL-2-induced LAG-3 overexpression (56). The addition of anti-FGL-1 mAb to 3A9 T cell lines resulted in positive regulation of TNF-α and an increase in IFN-γ levels, which restored T cell activation (56). These results suggest the importance of the interaction between LAG-3 and FGL-1 in T cell inhibition and tumor development.

### 3.5 TCR-CD3

Recently, Guy et al. have identified the TCR-CD3 complex as a potential novel ligand for LAG-3 (57) (**Figure 1**). Immuno-tyrosine-based activation motifs (ITAMs) in the CD3 complex are phosphorylated when the TCR identifies and attaches to the antigenic peptide-MHC complex (58). This phosphorylation, mediated by Lck (Src family tyrosine kinase), activates downstream signaling molecules such as ZAP70, LAT, and Syk, ultimately resulting in T cell activation, proliferation, and differentiation (59). This complex forms a tight contact area called the immune synapse. Guy et al. indicated that LAG-3 can bind to TCR-CD3 complexes and then migrate to immune synapse in CD4^+^ and CD8^+^ T cells in the absence of MHC-II (57). They suggested that conserved acidic tandem glutamic acid-proline repeats in the cytoplasmic tail of LAG-3 decrease the PH of the immune synapse, resulting in the dissociation of the tyrosine kinase Lck from CD4 or CD8 coreceptors and preventing TCR signaling and T-cell activation (57, 60). Workman et al. pointed out that the highly conserved ‘KIEELE’ motif in the cytoplasmic domain of LAG-3 is essential for its function (61). However, there is still no breakthrough in the study of this motif.

In summary, as an emerging immune checkpoint, the study of its interaction with ligands is still at an initial stage. Excluding MHC-II, the binding mechanism of other ligands to LAG-3 and the function of tumor immunity still need to be elucidated. Targeting LAG-3 and its ligands is one of the effective therapies to enhance anti-tumor immune responses and inhibit tumor growth. In-depth study of its interaction with ligands is essential to understand and elucidate the LAG-3-mediated immune inhibition.

## 4 LAG-3 as a potential prognostic biomarker in cancers

In the TME, expression of LAG-3, especially on TILs, is related to the inhibition of T cell proliferation and cell cycle arrest (62). This impairment of T cell-mediated anti-tumor immune responses suggests that LAG-3 expression levels in different cancer types correlate with clinical outcomes, thereby highlighting its potential as a predictive biomarker.

Numerous studies have elucidated that the increased expression of LAG-3 is able to improve the clinical outcome of cancer patients. For instance, Hu et al. conducted a meta-analysis and found that an increase in the density of LAG-3^+^ TILs was associated with improved OS in triple-negative breast cancer (TNBC) patients (63). In early-stage breast cancer studies, increased LAG-3 expression was also observed to be associated with longer metastasis-free survival (MFS) (64). In addition, Arimura et al. showed that higher LAG-3 mRNA levels were correlated with better OS in malignant pleural mesothelioma (MPM) patients (65). They also indicated that among 38 MPM patients analyzed by immunohistochemistry (IHC), those with higher LAG-3 protein expression levels showed better prognostic outcomes. Park et al. used monochromatic and multicolor immunohistochemistry to measure cell-surface LAG-3 expression in patients with gastric cancer. The results indicated that elevated LAG-3 expression was associated with improved prognosis in patients with stage II and III gastric cancer (37). Li et al. investigated the level of soluble LAG-3 (sLAG-3) in the serum of gastric cancer patients. They found that high level of sLAG-3 was associated with the increased frequency of CD8^+^ T cells and increased secretion of IL-12 and IFN-γ (66). Their findings collectively illustrated that LAG-3 is a potential independent prognostic biomarker for gastric cancer patients. Additionally, higher levels of LAG-3^+^ TILs were also associated with improved survival outcomes in CRC patients (67, 68). These findings highlight the potential of LAG-3 expression as a biomarker for favorable clinical outcomes.

On the other hand, some studies have also reported that LAG-3 can be a biomarker for poor prognostic outcomes in some cancers. For instance, the higher expression of LAG-3 and tumor-associated macrophages (TAMs) significantly increased in patients with Hodgkin’s lymphoma and were associated with shorter PFS and OS (69). Guo et al. showed that serum levels of LAG-3 increased significantly in patients with hepatocellular carcinoma (HCC), compared with healthy controls. High levels of LAG-3 in HCC patients are accompanied by cirrhosis, elevated levels of alanine aminotransferase (ALT) and aspartate aminotransferase (AST), and the progression of Barcelona Clinic Liver Cancer (BCLC) stage (70). These patients also had shorter OS and worse prognosis, suggesting that LAG-3 may also be a prognostic biomarker for HCC. In epithelial ovarian cancer (EOC), expression level of LAG-3 in TILs is negatively correlated with patient prognosis (62). The OS and disease-free survival (DFS) of EOC patients with high expression of LAG-3 were significantly decreased, suggesting that LAG-3 may be a biomarker for the EOC prognosis. In addition, LAG-3 mRNA expression in melanoma patients is thought to be correlated with tumor progression, and high levels of LAG-3 tend to represent poor DFS and OS (65, 71).

In general, some studies reported LAG-3 as a favorable prognostic biomarker, while other studies reported it as a poor prognostic biomarker. The differences in these studies could be attributed to different reasons. First, although LAG-3 normally inhibits the antitumor function of T cells, its high expression is often found on activated T cells, especially TILs. These activated T cells are able to maintain ongoing tumor surveillance and may represent a durable antitumor response of the immune system. Second, LAG-3 also plays a protective role in the immune system, inhibiting the excessive activation of T cells while preventing their excessive depletion. This balance mechanism helps to maintain immune activity in long term, which is effective in inhibiting tumor growth and spread. In addition, the co-expression of LAG-3 with other immune checkpoint molecules such as PD-1 has suggested in some studies that patients with high LAG-3 expression may have better therapeutic responses to immune checkpoint inhibitors, thus showing the potential for improved prognosis.

## 5 Targeting LAG-3 in cancer

### 5.1 Impact of targeting LAG-3 alone or in combination with other immune checkpoints

Several studies have shown that LAG-3 plays an essential role in immunosuppression and tumor growth, indicating that inhibiting LAG-3 could have positive therapeutic effects in cancer treatment (47). For example, *in vitro* studies have shown that inhibition of LAG-3 signaling in TILs from patients with melanoma restores the ability of CD4^+^ and CD8^+^ T cells to secrete IFN-γ, thereby enhancing their antitumor activity (25, 26). Furthermore, blocking LAG-3 on circulating NK cells from lung cancer patients increased cell cytotoxicity and IFN-γ levels (72). Maruhashi et al. suggested that the MHC-II and LAG-3 signaling pathways in Hodgkin lymphoma patients may be a valuable therapeutic target (19). The feasibility of this approach was demonstrated by the ability of IMP321, a soluble LAG-3 fusion protein capable of binding to MHC-II, to inhibit tumor growth *in vivo*, significantly activating CD8^+^ T cells, and extending PFS in patients with advanced renal cancer (73).

Relatlimab, the first LAG-3 mAb, has shown promising anti-tumor effects in chronic lymphocytic leukemia (CLL) by restoring the immune activities of NK cells and T cells (74) (**Figure 2**). Combined with lenalidomide (an immunomodulatory drug for multiple myeloma), it enhances IL-2 and NK cell-mediated antibody-dependent cell-mediated cytotoxicity (ADCC) (74). In addition, Thudium et al. reported that another anti-LAG-3 monoclonal antibody (clone C9B7W) effectively inhibited tumor growth in Sa1N fibrosarcoma and MC38 mouse colon adenocarcinoma models (75).

**Figure 3:**
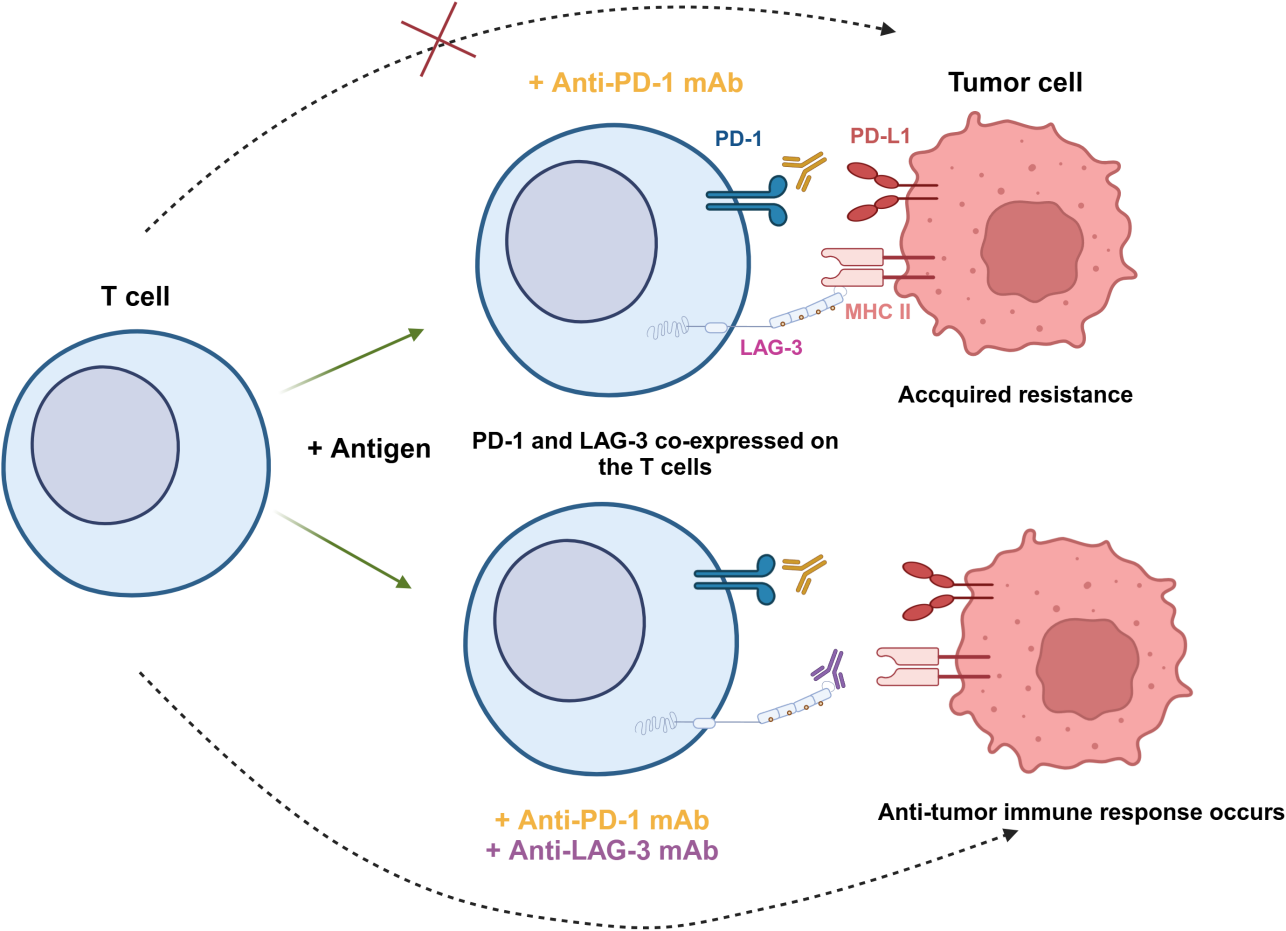
Synergistic effect of PD-1 and LAG-3 dual blockade in antitumor immunity. PD-1 and LAG-3 are co-expressed on the surface of T cells under antigen stimulation. When tumor cells express both PD-L1 and MHC-II, anti-PD-1 mAb is unable to completely block tumor cell escape, leading to acquired resistance (top). However, the combination of anti-PD-1 and anti-LAG-3 mAbs simultaneously blocks these two immune checkpoint pathways, restores the activity of T cells, and enhances the ability to recognize and eliminate tumor cells (bottom).

Although targeting LAG-3 alone has shown efficacy in restoring T cell function, many studies indicated combining LAG-3 and other immune checkpoints is more effective. Yang et al. performed a flow cytometric analysis of blood samples from patients with follicular lymphoma (FL) and found that LAG-3 was highly expressed on PD-1^+^ T cells in the TME (76). The ability of PD-1^+^LAG-3^+^ T cells to produce cytokines like IL-2 and IFN-γ, as well as cytotoxic molecules like granzyme B (GzmB) and perforin (PFN), was found to be lower than that of PD-1^+^LAG-3^-^ cells (76). Woo et al. showed in animal experiments that anti-LAG-3 and anti-PD-1 combination therapy increased the expression of IFN-γ in CD4^+^/CD8^+^ TILs and decreased the level of TNF-α^+^CD4^+^/CD8^+^ TILs (26) (**Figure 3**). This research demonstrated that combination therapy with anti-LAG-3 and anti-PD-1 slowed cancer progression by restoring and enhancing effector T cell populations in tumors and lymph nodes.

In addition, many studies also support the efficacy of combined blocking strategies in treating different cancer types. Huuhtanen et al. used single-cell RNA sequencing, T cell receptor sequencing (scTCRab-Seq), and other multi-omics techniques to analyze peripheral blood samples from melanoma patients treated with combination therapy with relatlimab and nivolumab (77). According to their research, LAG-3 was highly expressed on CD8^+^ T cells, NK cells, and Tregs in melanoma patients. Moreover, the expression level of LAG-3 was strongly linked to the degranulation activity of NK cells. Following anti-PD-1 and anti-LAG-3 treatments, NK cells degranulated and released cytokines that stimulated the proliferation of CD8^+^ T cells. Additionally, the combination therapy enhanced the cytotoxic properties of antigen-restricted T cells stimulated by NK cells and altered the expression profile of Tregs, compared to LAG-3 blockade therapy alone (77).

Andrews et al. found that CD8^+^ T cells lacking PD-1 and LAG-3 were unique at the transcriptional level in a melanoma mouse model, exhibiting a broad range of TCR clonotypes and abundant effector and interferon response genes, compared with CD8^+^ T cells lacking PD-1 or LAG-3 alone (78). These characteristics allow them to clear tumors more effectively and prolong the life span of mice (78). Ngiow’s study further showed that LAG-3 plays a crucial role in maintaining the persistence of exhausted CD8^+^ T cells (Tex) while generating a subset of CD94/NKG2^+^ Tex cells with enhanced cytotoxicity (41). However, Cillo et al. pointed out that although the combined blockade of LAG-3 and PD-1 can enhance the receptor signaling capacity and cytotoxicity of CD8^+^ T cells, it still retains the exhausted characteristics of Tex, suggesting that further optimization of future treatment strategies still needs to be considered (79).

Kureshi et al. recently examined the effects of LAG-3 and PD-1 inhibition in patients with advanced melanoma (80). They found that the combination therapy improved survival in advanced melanoma patients, with improvements in PFS and OS. Nevertheless, they also observed that patients with combined blockade still exhibited irAEs similar to those with PD-1 blockade alone, possibly due to incomplete blockade or partial overlap in cell types regulated by LAG-3 and PD-1 (80). In the combined blocking experiment of PD-1 and CTLA-4, Larkin et al. found that cancer patients in the nivolumab (PD-1 inhibitor)-plus-ipilimumab (CTLA-4 inhibitor) group showed more severe treatment-related adverse reactions (81). Therefore, the combination therapy with immune checkpoint blockade must be strictly regulated and controlled for different patients.

Collectively, these results demonstrated that inhibiting LAG-3 with other ICs, or the use of neutralizing mAbs against LAG-3 in patients who have become resistant to PD-1/PD-L1 therapy, might be a practical approach for overcoming tumor resistance to mAb therapies and providing therapeutic advantages to patients with acquired resistance. Up till now, LAG-3 blocking has demonstrated synergistic effects in various tumor models when combined with other immune checkpoint therapies, such as anti-PD-1/PD-L1 (26, 75, 82). These promising preclinical outcomes have led to the initiation of clinical trials to assess the safety and efficacy of LAG-3 inhibitors when combined with other ICIs.

### 5.2 Potential therapeutic strategies for blocking LAG-3 signaling in cancer

**Figure 4:**
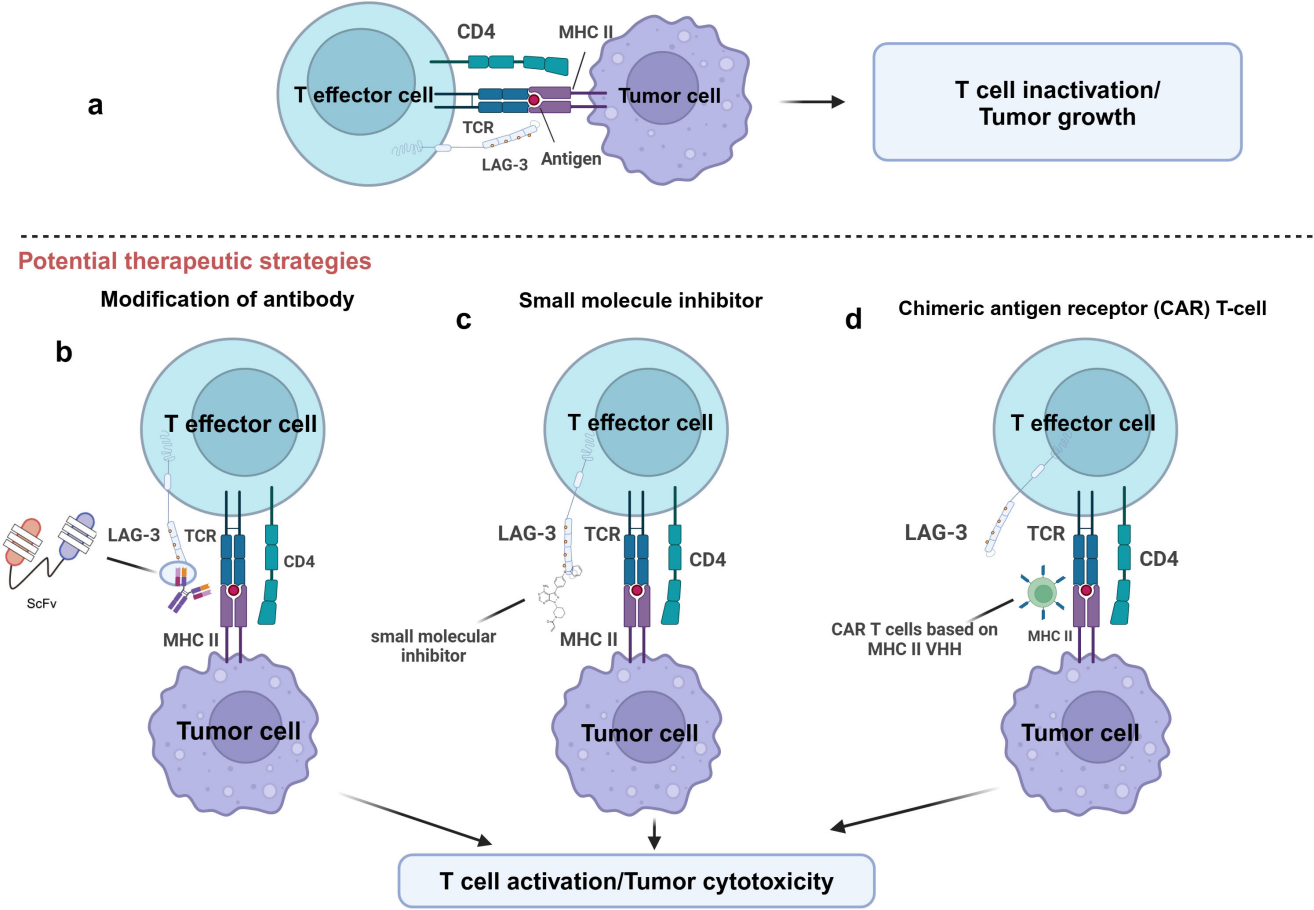
Potential therapeutic strategies for targeting LAG-3. This figure illustrates several potential therapeutic strategies to enhance T-cell function by targeting LAG-3. **(A)** LAG-3 binds to MHC-II on the surface of APCs or tumor cells and inhibits T effector cell activation, leading to T cell inactivation and tumor growth. **(B)** T cell activity is restored by blocking the binding of LAG-3 to MHC-II by modified antibodies, such as single-chain variable fragment antibody (ScFv). **(C)** Small molecule inhibitors block LAG-3-mediated signaling by interfering with its function to inhibit T cell activation. **(D)** LAG-3 VHH based chimeric antigen receptor (CAR) T-cell technology specifically targets and eliminates MHC-II expressing tumor cells. The goal of all these strategies is to restore T cell activity and enhance tumor cytotoxic responses by relieving the inhibitory effects of LAG-3.

Preclinical studies assessed the effect of inhibiting LAG-3 function on tumor progression by blocking the interaction between LAG-3 and its ligand using mAbs. However, the main limitation of mAbs is their difficulty penetrating the dense stroma of tumors, which limits their efficacy and affects clinical outcomes (83, 84). To overcome this challenge, researchers have developed antibody fragments with better penetration, such as single-chain variable fragments (scFv), which may improve clinical outcomes (83, 85) (**Figure 4**).

Several drug candidates targeting LAG-3 are currently in clinical trials for the treatment of chordoma, esophageal cancer, gastric cancer, and multiple myeloma (86). However, it has been found that these mAbs may cause systemic inhibition of LAG-3 in patients, thereby inducing irAEs, which emphasizes the importance of targeted drug delivery. Targeted delivery of antibodies to tumor sites has been proposed as a potential therapeutic strategy and been mentioned in the treatment of PD-1 and CTLA-4 (87). Based on this strategy, one potential alternative approach is to inject anti-LAG-3 mAbs directly into the tumor with therapies targeting tumor-associated antigens (TAA). This strategy can improve treatment specificity and efficacy, reduce systemic side effects, and maximize therapeutic benefit.

Despite these advances, mAb still faces resistance in some tumors. For instance, in NSCLC or melanoma, some patients do not respond to mAbs treatment or have disease progression after an initial response (88). In response to these problems, small molecule inhibitors are emerging as a promising alternative drug. In contrast to mAbs, small molecules with oral bioavailability enhance tumor penetrating (89, 90). Additionally, a small molecule is more easily optimized for pharmacokinetics, allowing for a flexible dosage regimen to mitigate mAb-related irAEs (91) (**Figure 4**). The development of LAG-3 small molecule inhibitors may offer a successful immunotherapy for managing certain solid tumors. Abdel-Rahman et al. used a combination of focused screening and “catalog search” to identify small molecules capable of inhibiting LAG-3/MHC-II and LAG-3/FGL-1 interactions (92). Early *in vitro* tests demonstrated that these small molecules could effectively block LAG-3 ligand interactions, indicating that they may be helpful as therapeutics (92).

Chimeric Antigen Receptor (CAR) T cell therapy represents another promising strategy. Xie and his team validated this therapeutic approach in a mouse tumor model (93) (**Figure 4**). They grew CAR T cells that recognize the VHH of PD-L1 and applied them to a B16 melanoma mouse model. The results indicated that these organized T cells significantly inhibited tumor progression and improved the survival of the mice (93). Notably, VHH-expressing CAR T cells were specific, active *in vitro*, and cytotoxic to PD-L1 in melanoma cells, M38 colorectal adenocarcinoma, and HPV16-transformed cell lines, with these properties being mediated through the secretion of IFN-γ. Based on this study, VHH CAR-T cells targeting LAG-3 ligands might be a viable and effective immunotherapeutic strategy. Compared with LAG-3, the ligands such as FGL-1 are expressed at low levels in normal tissues (56). Therefore, targeting ligands can help reduce systemic adverse effects, focus immune regulation on the tumor site, and improve the precision and safety of treatment. In addition, blockade of LAG-3 ligands can inhibit the immunoinhibitory effects of LAG-3 signaling pathway without directly acting on T cells, thereby protecting antitumor T cell activity and enhancing antitumor immune responses in TME.

## 6 Conclusion

**Figure 5:**
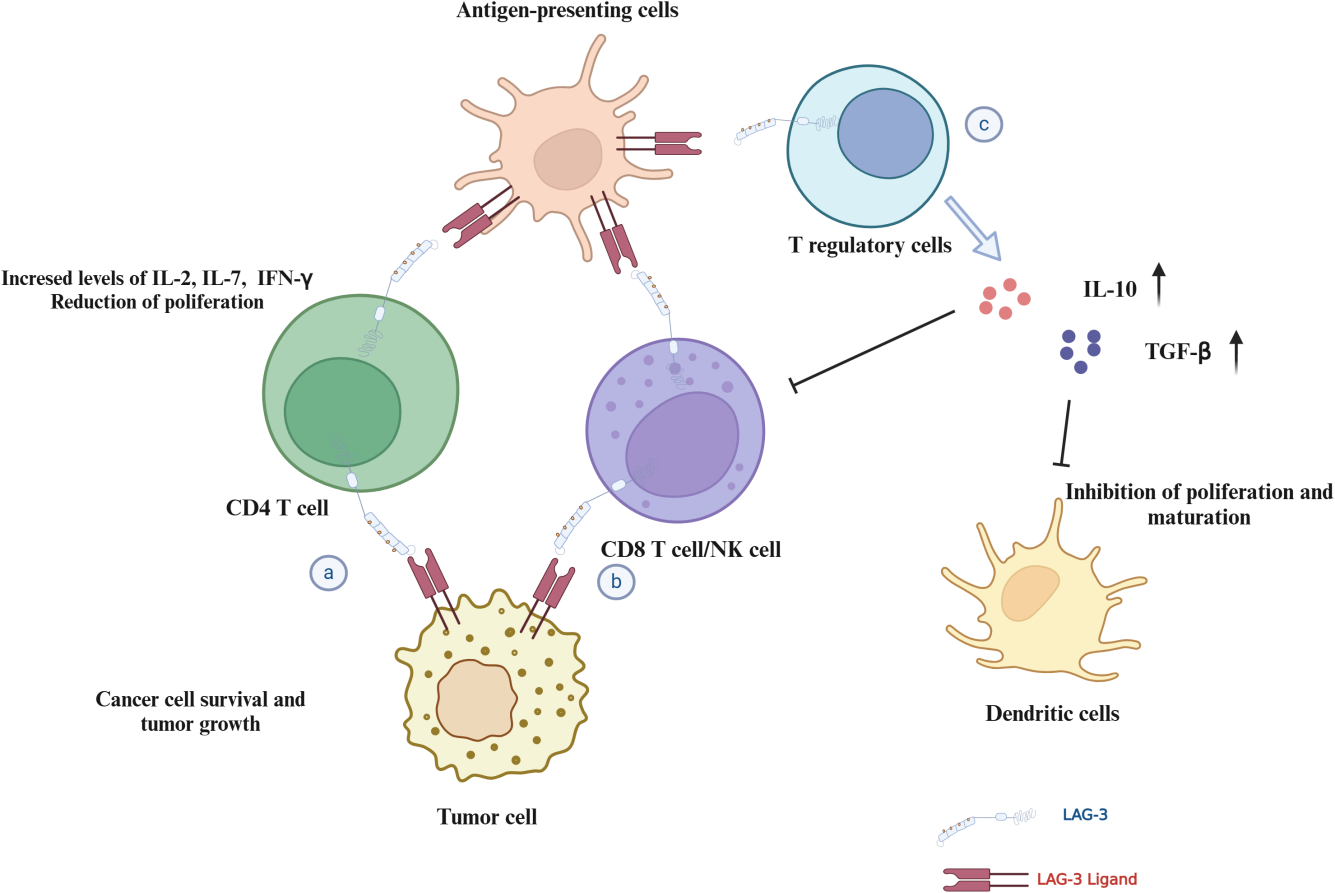
Mechanisms of LAG-3 in immune regulation and tumor microenvironment. This figure illustrates the multiple roles of LAG-3 in modulating the immune response and tumor environment: **(A)** CD4^+^ T cells: LAG-3 interactions with ligands inhibit CD4^+^ T cell proliferation and cytokine secretion, potentially supporting tumor cell survival. **(B)** CD8^+^ T cells/NK cells: LAG-3 interaction reduces proliferation and cytotoxicity of CD8^+^ T/NK cells in the TME. **(C)** Tregs and dendritic cells: LAG-3 interaction enhances the stability and immunosuppressive capacity of Tregs, while affecting dendritic cell maturation and immune stimulation.

LAG-3 has been identified as a critical immune checkpoint in regulating immune responses in the TME. As shown in (**Figure 5**), it acts by inhibiting the proliferation and activation of T cells, thereby promoting tumor immune escape and the progression of various malignant tumors. The interaction of LAG-3 with its ligands, such as MHC-II, Galectin-3, LSECtin, and FGL-1, highlights its potential as a therapeutic target and prognostic biomarker for cancer.

Numerous studies have revealed the therapeutic value of LAG-3. Blockade of LAG-3, alone or in combination with PD-1 inhibitors, restores T-cell activity and enhances antitumor immune responses, which is a promising strategy for overcoming resistance to conventional immunotherapy. Relatlimab, an anti-LAG-3 monoclonal antibody, has shown encouraging results, especially when combined with nivolumab, to enhance treatment in patients with advanced cancer.

Despite the therapeutic potential, the complexity of LAG-3 as a prognostic marker in cancer still requires further exploration. In cancers such as TNBC and gastric cancer, elevated LAG-3 expression is associated with improved clinical outcomes, and higher levels of LAG-3^+^ TILs are associated with prolonged survival. In contrast, in other cancer types, such as HCC and ovarian cancer, higher LAG-3 expression generally represents a worse prognosis. The duality of prognostic significance of LAG-3 suggests that focusing on LAG-3 expression alone may not be sufficient to predict the clinical outcome of cancer patients. More comprehensive analysis involving multiple immune markers may help better determine patients’ prognostic status.

Moreover, irAEs induced by blocking LAG-3 remain a significant threat to patients, especially when combined with other ICIs. Therefore, in the clinical settings, the control of irAEs is essential to ensure the safety and efficacy of the treatment regimen. Future research should focus on developing alternative therapeutic approaches, such as small molecule inhibitors and scFv. These therapies have better tissue penetration than mAbs, potentially enhancing their efficacy in the dense tumor environment. Small molecule inhibitors, in particular, may provide more flexible treatment options due to their oral bioavailability and adjustable pharmacokinetics. In addition, CAR T cell therapy targeting LAG-3 provides another promising avenue for cancer treatment, particularly in solid tumors, where conventional immunotherapy does not respond well.

Overall, LAG-3 represents a promising target in cancer immunotherapy. Its role in immune regulation and dual function as a prognostic biomarker makes it a vital focus for future studies. According to the Clinicaltrials.gov database, there are now more than 150 ongoing clinical trials targeting LAG-3, and more than 40 trials have been completed. Completed clinical trials using LAG-3 antibodies in monotherapy and in combination therapy are shown in **Table 1**. Exploration of LAG-3 targeted therapies, particularly in combination with other ICIs, can potentially improve clinical outcomes, particularly among patients with refractory or advanced cancers.

**Table 1:** Completed clinical trials using LAG-3 antibodies in monotherapy and in combination therapy.

Name	Combined with	ClinicalTrials.gov ID	Phase	Tumor type
Urelumab	**-**	NCT02658981	I	Glioblastoma
Sym022	**-**	NCT03489369	I	Lymphomas
HLX26	**-**	NCT05078593	I	Lymphoma
IMP321	**-**	NCT00351949	I	Renal Cell Carcinoma
IMP321	**-**	NCT00349934	I	Metastatic Breast Cancer
INCAGN02385	**-**	NCT03538028	I	Cervical Cancer
Relatlimab	Nivolumab (PD-1)	NCT03743766	II	Melanoma
Sym022	Sym021 (PD-1)	NCT03311412	I	Lymphomas
Relatlimab	Nivolumab (PD-1)	NCT03662659	II	Gastric Cancer
TSR-033	Dostarlimab (PD-1)	NCT03250832	I	Advanced Solid Tumors
Relatlimab	Nivolumab (PD-1)	NCT03623854	II	Advanced Chordoma
LAG525	PDR001 (PD-1)	NCT03365791	II	Hematologic Malignancies
REGN3767	REGN2810(PD-1)	NCT03005782	I	Advanced Malignancies
